# Fever

**Published:** 1873-08

**Authors:** Thomas A. Cooke

**Affiliations:** Louisiana


### SOUTHERN MEDICAL RECORD.

VOL. III.—AUGUST, 1873.—No. 8.

### PART I.

### ORIGINAL COMMUNICATIONS.

### FEVER.

BY THOMAS A. COOKE, M.D., OF LOUISIANA.

In considering the subject of fever, it is important to hold in mind the fact that the human body, however divided by the physiologist and anatomist into tissues, systems and organs, is yet a unit. The final division is into solids and fluids. The solids depend upon the fluids for the retention of healthy action, and the condition of the latter is immediately affected by a failure of the former to receive and elaborate the elements of nutrition which it furnishes. And thus it happens, from a modification of organic action, that there ensues either an increase or a diminution of the depurative action of the organs: in some cases sustaining the blood with elements indispensable to life—in other cases furnishing no elements, and thus aiding in rendering the blood no longer a life-sustaining fluid. The movements which constitute health—the result of the unimpeded power of the force of life—are inseparably united, blending and acting together in virtue of the truth of the corporate character of all the most important organs embracing blood and nerve substance. A composite organ loses at once its integrity when a component tissue or system is absent or defective, whether resulting from external or internal causes; and just in proportion to the injury of tissue, or of the functional action of the system, will that organ of which either may be an essential part become affected, perverted, diseased.

The blood at the present time seems to be regarded, not only as the chief channel through which disease is imparted, but as the seat of the system affected in a large number of complaints. We read in every book, we hear the statement constantly made, that it is a blood disease—a blood poison.

No one will deny that this fluid does play a most important part, either primarily or secondarily; that is, not only as the system through which the morbific cause is conveyed, but from the vitiation of its nature resulting from disease. But, is it not true that the presence of deleterious principles in this fluid—and, in all probability, likewise the absence of important elements—may not prove to be incompatible with health? Do we not witness in every epidemic, whether of infection or contagion, facts which demonstrate the perfect compatibility of poisons in the blood with a continuance of health? No one conversant with plague and pestilence, spreading through a whole community, but has seen in many instances the utter inocuousness of the offending cause. The inherent preservative power belonging to all the organs expels from the body the deleterious principles which it is conceded have entered into the blood. The poison introduced into the sanguinious system is excreted, and no recognizable symptoms of disease are seen until the forces of health succumb, and the organs are rendered unable to resist the morbific influences of the deleterious elements in the blood. In the extent of the enfeebled or interrupted action of the eliminative functions, we read the extent of the involvement of the organs, and the disease resulting will always depend, as to its violence and nature, upon the special character of the poison and the imperfect nutritive actions consequent upon the impairment of the forces of health. When the organism has been subjected to depressing causes—as poverty, insufficient or unwholesome food—all those influences which slowly enfeeble and derange the animal economy—as, for example, a deranged constitution of the atmosphere—the forces of health, after a fruitless contest, will be affected in proportion to the nature and continuance of the morbific influences to which the system is exposed. But, admitting that the blood and nerves first introduce the causes of disease—are the first systems which experience the presence of poisonous influences—it does not, in my judgment, follow that these systems are to be regarded as the chief seats of the disease. Remove the poisonous influences, place the patient beyond the sphere of their action, and then the blood will prove to be dependent upon the restoration of digestion, and of the eliminative functions, for the resumption of a healthy condition; in other words, restore these functions, and health invariably returns. Or, if this fluid remains in a morbid state after the removal of the external influences, it must, of necessity, result from an inaction or impairment of nutritive organic actions, especially of such organs as are charged with the office of sanguification—or from what seems to me to be the fact—that is, a general impairment of molecular actions.

On the supposition that fever has arisen without a foreign cause—and this idea is not only conceivable, but would naturally be suggested, for the reason that the common causes to which it is attributed are impalpable, mysterious and inexplicable, asserted and rebutted by equal authority—the inference is clear that the affection springs from external influences affecting organic actions. In this supposed case we can look only, for an explanation, to the perversion of those innate organic actions, to a derangement of the vital chemistry of the parenchyma of all the organs, including all the tissues and systems constituting, by an inseparable union, the human body. It matters little whether the nerves have been first impressed, or whether the element of disease be floating through the blood, it is to the organs, consisting of these systems and of all tissues respectively belonging to them, that we must look for a solution of the phenomena of fever; for nervous derangements oftentimes speedily cease, and the blood may be the constant recipient of morbific causes which, being thrown off, are impotent in the causation of fever. But so soon as these organic actions are affected, with their first modification, attended with diminished or increased action, will symptoms of disease show themselves. The inseparable union which exists between all the tissues of the body causes a reciprocity of condition between them, so that one can not become permanently deranged without involving all in disease.

After a certain period of exposure to the causes of fever, the forces of health succumb, the whole body becomes affected, the organs all undergo serious modifications in the discharge of their functions, and the blood, whether or not there be in it the cause of fever, no longer derives from the waste of the body the necessary materials for its proper preservation as a life-sustaining fluid.

In one form of fever we behold, as it were, a melting down of the system, and in another the symptoms announce in some cases an impairment, in others more violent, fearful suspension of the remote organic actions. These phenomena must, it seems, depend upon the nature of the poison which affects the whole system. Thus, in typhoid fever, from its commencement a great waste of the body constitutes one of its most important characteristics. The loss of weight is known in many cases to exceed daily the weight of food the patient was accustomed to take in health. During the continuance of the fever this waste of the body goes on, for weeks, for months; and for this long period the only support of life, in the prostration of the digestive organs, is found in aliments furnished from the body of the patient. From every organ and tissue of the body elements are respectively furnished, to the extent of preserving the blood as a life-sustaining fluid, of preventing the disorganization of solids, of aiding in its turn the preservative forces of life, until the recuperative energies, the forces of health, no longer impeded by the morbific cause that had been fastened on the system, resume their power and insure convalescence and health. In the absence of food from without, consequent in many cases upon the complete prostration of digestion, it is clear that life could have been sustained in no other way. In this severe and protracted affection, against which it is doubtful if the physician possess a single weapon, offensive or defensive, Nature furnishes from her own storehouse aliment, and perhaps medicine, by which death is warded off and safety finally secured. Notwithstanding the continuance of fever for weeks and weeks, and in many cases the rejection of all food—during a progressive waste of all the solids—we see in most all cases in which active medicines have been withheld, more or less healthy passages from the bowels; we witness a continuance of all the important secretions, especially of urine and of bile. In this protracted disease, whence come the elements from which the kidneys and liver elaborate their respective secretions? And here, though perhaps out of place, may be asked, whence for days the source of excessive secretion of bile in what we call bilious fevers? As a general rule, health and emaciation proceed *pari passu*. It is not necessary to pursue these reflections further. It appears, then, just to presume—indeed, to state as a proposition—that in all severe fevers, (I reason from a survey of the symptoms of all uncomplicated cases of bilious, typhoid and yellow fevers), that a continuance, and, in cases of a suspension, a more or less speedy resumption of the molecular organic actions is necessary to furnish materials of heath to the blood, and is indispensable to the recovery of health.

Let us now consider the progress and leading symptoms of a bilious autumnal fever. The stages of this affection have been divided into the cold, hot, and sweating—the remission or intermission speedily following the last. During the cold stage, the energies of the general system are more or less prostrated; the blood leaving the surface and circulating sluggishly in the deep-seated organs. In the second, or stage of reaction, there is ordinarily great heat of surface, high arterial action, with other local and general symptoms. Draw blood from the veins in a high fever, and its arterial color, always seen in a degree more or less marked, and accompanied with severe suffering, announces that there is an impairment, more or less serious, of organic actions; that, for the time being, the all-important vital process of supply and waste has been rendered feeble or inactive, and that the forces of health, life itself has been attacked in its very citadel. This state of things, plainly seen by the eye, it will readily be acknowledged, would be perilous, unless it speedily yielded to the restorative powers of Nature. And, accordingly, we have full evidence that this restorative power is renewed during the decline of the hot, and is fully established in the sweating stage, as is shown by the increased darkness of color of venous blood, by the shrinking and waste of the solids. Now we see the evidences of a more or less excessive depuration from the whole body, and, often without the aid of medicine, Nature has sufficed to expel the offending cause and to restore health. At the height of this fever it would appear as if there was an unbroken continuity between the arteries and veins, for in many cases there is small difference in the general aspect of the blood in both systems of vessels. In fact, the body appears bloated, the solids swollen, all appearing to suffer an universal irritation, and the blood is no longer deoxydized, because of the absence of carbon and its compounds.

This fever, though seen in all seasons of the year, yet belongs especially to summer and autumn. The speedy recovery of cases which we see ushered in with extreme severity, shows that the causes of the affection, as well as the physical modifications which ensue, are entirely different from what we witness in typhoid or yellow fever. It is this latter affection which fully illustrates the peculiar physiological derangement which constitutes the essential physical condition upon which fever depends, and which it appears to me is indeed the disease itself. Yellow fever is acknowledged to be a disease of one paroxysm, without remission or intermission. Some writers speak both of remittent and intermittent forms of the affection. They must be regarded as exceptional cases. I once saw a case, in the midst of an epidemic, of a violent intermittent fever, in which, during the febrile paroxysm, the symptoms completely resembled those of yellow fever, and I felt authorized to assure the patient after his recovery that he might consider himself acclimated. In the same house in which this man was sick there was another patient which had come to town to be treated for an intermittent fever, from which he had been suffering for some time in the country. The second paroxysm in this case proved to be one of pure yellow fever, which, after a period of about three days, was followed by a collapse with black vomit and death. The intermittent invariably runs into yellow fever on exposure to the cause of the last. The exceptions do not in any way affect the proposition that yellow fever is a disease of one paroxysm. Unlike the bilious, it presents no remissions or intermissions. Without premonition it announces itself by a chill, or rather a rigor, which stage varies in duration from a few minutes to an hour. In the reaction we often witness fever in its highest grade. The pulse reaches one hundred beats in the minute, more or less. The heat of the body is extreme. The blood courses through the vessels with such rapidity as to threaten exudation through the pores of the skin; the face is suffused and swollen; the eye red, inflamed, and very painful; the brain feels oppressed with a heavy weight, and, in connection with back and bones, is the seat of intense suffering. At the onset, some yellowish fluid may be ejected from the stomach, but it soon ceases, and matters thenceforth discharged from the stomach—and also, after the first few passages, from the bowels—show an entire absence of bile. More or less nausea is apt to continue, but the matter vomited consists entirely of the ingesta and some mucosities. The bowels, easily moved at first, soon manifest the absence of watery or serous effusions. The kidneys gradually lose the power of secretion until, sooner or later, ensues a suppression of secretion of urine. Generally, after the first few hours of fever, we see more or less perspiration, active at the commencement, but gradually assuming the character of a passive exudation. The face and body present a swollen, bloated aspect, which seems to become more manifest as death approaches. But the most remarkable characteristic of the disease is the change in color and in the constitution of the blood. From the beginning, the venous approaches toward the hue of the arterial, and very soon suffers a great impairment of the property of coagulation. As the disease progresses, the blood becomes more and more pale, and coagulation more and more imperfect. Such a condition presenting a forcible contrast with what we witness in the period of decline of fever in the remittent and intermittent forms—the one accompanied with a turgescence of the body and diminution of all the secretions, the other with an evident waste and with free secretions; the one hastening to a fatal issue, the other to recovery—such phenomena attest in the one a more or less complete arrest, in the other an increased activity of all the processes of depuration. Sometimes in the last moments of life, from the lips, mucous membranes, from abraded surfaces, there is an exudation of a fluid of a sooty blackness, and this may occur without the ejection of black vomit. This observation leads to the inference that, in such a case, the blood has received from some source acids, which, on admixture, always blackens it. It is self-evident that such acids are furnished by the system itself. The blood, as has been said, presents a marked increasing vitiation from the incipiency of the attack throughout the course of the disease. Whence this extraordinary result? At the commencement it resembles, in its aspect, that which is seen in common ardent fevers, during which it is universally admitted there is more or less lethargy of elimination. In the one case, with recovery, the venous is more dark than its natural hue; in the other, with the decline of fever, we witness no such change—indeed, in the earlier stages of yellow fever, resumption of its venous color is hailed as the harbinger of safety. Can it be that the increasing vitiation of the blood is due to the original poison absorbed into it, conceding that the sanguineous current is the conduit through which the poison first circulates; or are we not justified, by analogy, in the conclusion that the continued depravation of this fluid results from an impairment or arrest of depurative organic actions? Inspection after death shows a reddish or pale-red hue of the blood, semi-coagulated or flaccid. The liver is seen to be of a rather bright brick-dust color, more or less friable and containing no bile, and the gall bladder distended with a greenish or dark-colored matter, like tar. No sign of inflammation or structural change of any organ is, as a general rule, discoverable. The spleen and kidneys present the same brick-dust color, not only of their substance, but of the little blood which may be pressed from their partially dried-up tissues. Some mucosities are found in the alimentary canal. The patient rarely survives the fifth day—frequently death takes place many hours sooner. And now, viewing the dead body, nothing like wasting, in those simple cases which we are considering, is seen. On the contrary, the body looks full, and we are at once impressed with the fact that there has taken place, during the progress of the disease, no deperdition of the solids. In most all cases, as soon as life ceases, simultaneously with the cessation of the heart's action the whole body becomes suffused with a pale-yellow color. In some few instances a jaundice is seen before death, but very rarely in the cases we are considering, where the issue is death within five days. This phenomenon—strikingly illustrative of yellow fever—in connection with the absence of bile in the liver, gall bladder and ducts, shows that the coloring matter of the bile had not been separated from the blood during the progress of the disease, and *a fortiori*, that at no time from the inception to the end that the liver had been performing its essential function. It is unnecessary to present a more extended account of the deeply interesting phenomena of this affection to justify the statement that, notwithstanding the part the blood may have played in the causation of the fever, and also its subsequent vitiation, that the essential object of all medical treatment is the restoration of the eliminative actions, the renewal of vital chemical movements in the parenchyma of the organs.

In illustration of this statement, much light is derived from the history of all essential fevers. In cases of the typhoid, unaccompanied by a perversion of the nervous centers, or engorgements or inflammation of some one or more organs, a restoration to health, with little or no medical interference, will be witnessed about the eleventh, thirteenth or twenty-first day. So, also, in bilious fever, we predict a speedy recovery when we witness a free performance of the functions of secretion and of excretion. We hail with satisfaction the secretion of bile. The absence of this secretion, with or without diminished action of the kidneys, is ever recognized as a very unfavorable symptom. It is an evidence that the whole system is deeply stricken by the causes of the disease, that the depurative processes have been impaired or suspended; or it announces the danger which accompanies the retention within the blood of the elements of bile, and of other suppressed secretions; or that in the diseased state of the body, the elements necessary to the eliminative processes no longer exist in the blood. Regarding the liver as simply inactive, we behold a secondary condition, becoming in its turn a fearful cause itself of disease—a cause only to be removed by a resumption of biliary secretion.

In the history of yellow fever we have a demonstration of the views which we are endeavoring to explain. We see this disease presenting every grade, from the mildest self-curative form to that of the most appalling character. In the midst of an epidemic we shall often see the simplest form of the affection in the young babe, who presents a small febrile disturbance, sometimes not strong enough to arrest the attention of its mother, and in a few hours the child has become acclimated. In the matured subject, the symptoms of a favorable prognosis, the precursor of returning health, are recognized in the continued activity of the secreting organs, and could we be certain of the continuance of this activity, we would be justified in withholding all active medicine. But, inasmuch as we cannot, from the symptoms existing, predicate a continuance of the restorative movements, we are constrained to resort to remedies we deem best adapted to the case. Every physician of experience has seen cases like those alluded to, in the treatment of which he has discarded all active medication, and contented himself with simple adjuvants. But these, unfortunately, are only exceptional cases. They serve but to enhance the painful anxiety which we shall, in the present uncertainty of our remedial resources, feel in contemplating the generality of cases, for in them we witness the greatest intensity in the contest between the forces of health and the morbific influences acting upon the parenchyma of the organs. And notwithstanding a general similarity of symptoms, a remarkable uniformity in the expression of suffering nature, yet we witness in the different grades of the intensity of the disease symptoms which, though analogous in nature, present marked modifications. We can not but, in many of these cases, appreciate the fact that the nutritive actions have been stricken into a state of serious inaction or lethargy; that the blood in a half-dissolved condition can not, unchanged, much longer sustain life, but that this blood still desires some renovating elements from the system, and that at this critical period the safety of the patient, in the almost utter impotency of medicine, depends entirely upon the exercise of the force of health, however feeble it may be in furnishing from the laboratory of Nature elements to sustain its vitality, or to counteract those of a deleterious nature which may exist in it. In these cases there exist a profound derangement of all the organs; we see that a contest is waged for life or death, and in proportion to the predominance of one power over the other will result an impairment or exacerbation of the symptoms, and often for hours and days, in the equality of the powers engaged in the strife, we shall witness no change whatever. As might justly be anticipated, from such a condition of the system, whether the progress be towards death or recovery, we shall see a long array of ghastly symptoms—a swarthy, dirt-colored aspect or complexion; extreme restlessness, or stupor, or coma, or delirium; blood exuding from the mucous membranes, from blistered or abraded surfaces, staining the gums, lips, bed-clothes, etc. The allusion here made is to those cases in which, after the fourth or fifth day, constitutional disturbances with considerable febrile excitement occur, and which are examples of a secondary or consecutive disease, most frequently fatal in grown persons, but often in children ending favorably. In these cases, the physician is painfully sensible of his want of resources—for, with the exception of the administration of the hydrate of chloral, or some soothing medicine, and the timely use of a little castor oil, he has acted the part of a spectator. But Nature yet in many cases conquers. The organs slowly regain their functions—the machine is again put in motion.

There can not be offered to the contemplation of the reflecting physician a picture of disease more mysterious than that which is often seen in the last hours of a fatally-progressing collapse in a simple case of this disease. Some three days of an intense but gradually declining fever are succeeded by this stage of collapse. The general aspect of the patient will inspire the inexperienced with no alarm. The pulse is equable, full, and sometimes slower than in health. The disease seems to have departed, leaving the patient in a state of lethargy. When aroused, he declares that he feels no uneasiness—that he feels well. He will converse with perfect intelligence, but will speedily fall into an apparently refreshing sleep. In such cases I have seen individuals in whom the last spark of life was nearly gone out—in whom existence was sustained only by some unseen internal stimulus—indulge in merriment and song. I have seen the intelligent patient, after wiping from his mouth a sooty black matter, at once sink into the arms of death, though a few moments before he indulged the hope of a speedy convalescence. Such a scene is never witnessed in any other disease. We do not perceive the commencement of the fatal issue in the brain or nervous system; indeed, this whole system derives from the internal stimulus, which must permeate the body through the blood, an influence preservative of the mental powers; nor is any incipient dissolution to be traced to the lungs, the heart, stomach, or to any one organ more than to another. In a state of placidity of both body and mind, the patient without premonition will eject without effort a large amount of black vomit, or he will rise suddenly from the bed and almost instantly sink into death. The blood, notwithstanding a protracted collapse, retains the yellow color, and there is no emaciation of the body seen, except in some cases of protracted black vomiting. But in all cases of recovery, a waste of the body—the prophetic announcement of safety—is always seen, and sometimes to an extreme extent. With the commencement of convalescence, an extraordinary debility of the whole muscular system always manifests itself. The patient after a few days' sickness is unable to rise in bed. In some cases we seem to witness a transition from death to life, and we may justly look for a solution of this debility in the fact that the organs have for days been obstructed with pent-up matters—secretion and excretion having apparently ceased; that they have been deprived of nutrition for days; that at this period they receive a blood unprovided with nutritive atoms, and reduced in power as a life-sustaining fluid by losing the very element of stimulation which, to a certain extent, was necessary to the continuance of life, and for a time by the absence of restorative principles from without. It seems to me, in this condition of things, we may account for the great debility which has ensued, and for the imperatively demanded injunction to the attendant to give the patient only the lightest and most digestible article of food, and in the smallest quantity. Indeed, at this period (of what we may call convalescence), imprudences the most trivial have often led to fatal results.

I have no doubt indulged in a tedious relation of some of the remarkable phenomena of yellow fever, but I have been led to do so for the purpose of placing in bold relief the facts—which speak with stronger force than any poor explanatory words I might employ—facts upon which rest the pathology of fevers which I have been endeavoring to explain; and, moreover, by a desire to place before the reader many mysterious conditions of the system, which he will not meet with in books or monographs; and, I will add, also by a desire to show the reasons for the treatment I shall recommend. It is my wish to invoke especial attention to the condition of the organs in all essential fevers. It seems to me idle to discuss, in the consideration of uncomplicated cases, presumed aberrations in the constitution or functional action of the sanguineous and nervous systems. They are all involved in disease. They enter into the composition of every organ, and when the causes affecting them have continued to act, or to accumulate, they affect the forces of health; the movements of life, which, innate in all the organs, and which, in their unimpeded exercise, constitute perfect health, become stricken or impaired in power; and we infer that the disease ensuing will derive a character from the nature and quantity of those causes which have been introduced into the system. In the eruptive fevers, though we may admit that some peccant matter has been absorbed, and indorse the ingenious fermentation theory of Liebig, yet, in many of these affections we see in the perversion or inaction of organic movements facts which sustain the views entertained.

With the exception of certain neuralgias, which are relieved or benefited by special remedies, in the treatment generally of chronic complaints, in cases of enfeebled constitution, and of impoverished blood, tonics and stimulants and proper diet are mainly relied on, and the physician is impressed with the fact that restoration to health depends upon the resumption of natural organic actions indispensable to the renovation of the blood. After a protracted survey of physical perversion in typhoid fever, and the conflicting systems of treatment, he has come to the conclusion that he possesses no reliable medicines; that in the ordeal to which the patient is subjected, marked by progressive emaciation, continued excitement, and various symptoms indicating a profound vitiation of the whole system, he feels that he is limited to such remedial agents as are calculated to strengthen the resources of nature, to promote digestion, and to supply aliment. A blood poison may exist, but he feels his incompetency to neutralize or expel the peccant matter, and he is forced to acknowledge that the salvation of his patient depends upon this blood, depraved as it may be, and upon the continuance of the health-power to supply from every tissue elements which, combined with oxygen obtained from without, secure vital sustenance to the blood until Nature expels, not from the blood, but from the organs, the morbific influences which have been acting on the organs. Bilious fever is attributed very generally to miasmatic poisons. To me, it appears that heat, moisture, the vicissitude of temperature as is seen day and night—circumstances which affect, no doubt, the constitution of the atmosphere—are, in our climate, sufficient to bring about the peculiar disturbance of the system in this disease, to suffice to suspend or impair vital organic actions to the extent of exciting fever, which the health-power institutes for the purpose of restoring the natural state of the organs. Whatever cause engenders this disease, in its natural progress we witness the remarkable occurrence of periodicity. Formerly, before the powers of quinine were fully appreciated, this affection was prolonged for days in many instances; but of late years it appears to be under the control of this medicine. In very severe cases, accompanied with intense actual excitement, a moderate bleeding would be proper, to be followed immediately by two or three grains of opium and fifteen to twenty-five of quinine, to which it will be well to add from five to ten grains of calomel and two or three of ipecac. There results very speedily copious perspiration, an assuagement of pains and suffering, and we see what is termed an abortion of the fever. If the bowels are not moved within a reasonable time, a mild purgative is demanded, and it will be prudent to administer about ten grains of quinine an hour or two previous to the expected return of the fever, and this for two days. We know that this disease, if neglected, will generally return, and with symptoms aggravated, or which inspire alarm. With each returning paroxysm we realize the fact that the powers of health, though partially restored during the intermission, have become more enfeebled and incapable of throwing off the morbid influence; that local engorgements ensue, generally arresting the intermission, and sometimes supplanting the original affection; and that a change of treatment becomes necessary. In the case first cited an alarming disease, with menacing symptoms, has speedily ceased; the oppression of the organs, whether a poison circulates or not in the system, has passed away; the machine, apparently profoundly injured, has resumed, as by a charm, its harmonious action. After the fever has commenced to decline, it would not be prudent to adopt this treatment, in which case we must resort to measures calculated to promote all the processes of elimination, and by the use of quinine, as soon as possible, to prevent a return of the paroxysm. In country practice we are generally driven to this course.

With regard to the treatment of yellow fever, I am prompted, as the season of the year is rapidly approaching when the disease can alone prevail as an epidemic in this region, to present views which are sustained, according to my judgment, by its pathology, and which an extensive experience justifies me in offering. A disposition to theorize belongs to the nature of man, and in those cases in which the facts upon which theory may rest are not, as is the case generally in those of a medical character, susceptible of positive demonstration, all that can be expected is an approach as near as possible to useful practical results. It is certainly not flattering to the pride of the profession to be forced to acknowledge that, though more enlightened views have caused the abandonment of many theories, yet the practical lessons resulting from them have not been condemned or fallen into desuetude. We are all more or less governed by our views of pathology, and therefore we should, in the discharge of our duties, endeavor to study Nature, and to comprehend her mysteries, by the use of all means within our reach. Let us remember that in this disease the fever is intense, the heat of the body extreme; that the organs are blocked up; that the arterial blood courses without apparent interruption through the tissues, the capillary structures, and is received by the veins, which carry it back to the heart in a condition very similar to that with which it first departed on its circuit. It is propelled by the heart with great force and rapidity. In the bilious fever, Nature soon resumes—in the typhoid she never loses—those molecular movements which can not long be suspended without a fatal result. In yellow fever the very opposite is seen. The health force is from the beginning profoundly stricken. Functional organic action becomes more and more depressed, until, after no long period, there is an arrest of secretion and excretion. For some three days the excitement may continue. The highly arterialized blood seems to intoxicate the organs. There is some element in it which stimulates and may preserve structural integrity for some four or five days, but which affords no sustenance. Life can not long endure under these circumstances. We refer back to a fuller account of symptoms—to the appearance of the corpse—to the developments of the scalpel. This is certainly no disease for a milk-and-water or expectant treatment. The question goes beyond that of simply aiding Nature to relieve the system of an oppression. In a large majority of cases, luckily, Nature suffices to effect a cure. But the disease claims as victims about twenty per cent. of the cases, and amongst the latter are embraced too often those who are nearest and dearest to us. When we interrogate Nature, the answer she utters in the symptoms of the disease—in the extraordinary progress which the affection of one paroxysm makes—the inefficiency of medicines, which, under apparently similar circumstances, produce their desired results—in the peculiar aspect of the body—in the empty and shrunken bladder—in the contracted bowels, sometimes rendered impervious—in the bright-red color of the important organs—in these and other strange conditions Nature, in her answer, cries for relief from the burthen which oppresses her, and which has arrested or limited the vital actions of all the organs. Every moment is precious. Very soon the recuperative energies of the organs may be lost, never to return. In a few hours the patient may pass from the stage of extreme excitation to a fatally progressive collapse. Hence the importance of the immediate employment of heroic measures. Bleed from the arm freely—in the strong, lavishly. In no other disease, essential or inflammatory, can blood-letting be carried to so great an extent. Bleed, then, until the general excitement is lessened, suffering mitigated or removed, regardless of the quantity of blood drawn—governed by the effect produced. Then immediately follow the depletion by a combination of quinine, grains fifteen to twenty-five; powdered opium, two to three; calomel, five to ten; and of ipecac, two to four. There succeeds very speedily a subsidence of fever, with an assuagement or removal of suffering. Without repeated foot-baths, without oppressing the patient with blankets, perspiration streams from the body in rivulets; refreshing sleep ensues; there is free action from the bowels, and the patient is already, in most cases, out of all danger. It is rarely necessary to repeat the medicine, but it should be if its constitutional effect is not produced in an hour. In our common bilious fevers, a collapse with congestion has been known to follow blood-letting. In yellow fever such result never occurs. In the epidemics of New Orleans, just preceding that of 1853, the good effects of quinine and opium, employed in high doses, inspired many physicians with the belief that they had discovered the remedy which gave them full power over the disease. The epidemic of 1853 dissipated this pleasing illusion, for in the fever of that year there was such a tendency to cerebral disturbance, which seemed excited or increased by the medicine, that it was withheld or rarely administered. I can not but express the conviction, from an extensive observation of the disease this year, that an early resort to venesection would have removed the obstacle to the employment of the treatment. In this epidemic, blood-letting freely practiced at the onset of the attack proved so salutary that I did not often give the quinine and opium. Certainly, should this treatment, even after bleeding, tend to cerebral engorgement, it should not be used. In past times, blood-letting was regarded as essential in the treatment of this fever. Why has it been generally abandoned? The answer is found in the fact that when practiced after the first hours of, or late in the disease, as has invariably been done by those who condemn it, it is not only unproductive of good, but is promotive of evil. In those cases of one or two days' duration, though accompanied with much excitement, and in which Nature is slowly but successfully contending against the disease, blood-letting, by enfeebling the organic energies, will militate against recovery. The disease is rapid in its progress. Hence, if energetic measures must be employed, they should be in the beginning. It is impossible to establish the period, in a disease which proves fatal in from two to five days, an affection of one paroxysm, beyond which our remedies will prove inefficient. We shall often witness two cases, presenting very similar symptoms, and in one recovery, and in the other death, ensues. Surely, we have no right to boast of the efficiency of our remedies—to say that with some nitrous drink, gum water, or soda and morphine, or porter and champagne, we have cured the one patient. The homeopathist boasts, and it appears with some reason, of his success with his infinitesimals. In practice, the experienced physician condemns active medicines. The question is, can we lessen the mortality, which is sometimes over, sometimes under, twenty per cent. of the cases. Unfortunately, the physician in many instances does not see his patient until long after the time for the employment of active measures. If it be true that the disease is amenable to such measures, employed in the first hours after the attack, but that after this period the organs are so disordered as to lose their recuperative energies—that the blood is exhausted of elements necessary to secretion—no opprobrium should be attached to the profession where medical assistance has been delayed until the results above mentioned have occurred. In this disease, above all others, the people should be warned of the importance of early medical aid; and we express, unequivocally, the conviction that success will follow, as a rule, the treatment recommended. Let me cite some cases illustrative of the precept I desire to inculcate.

W. Allen, a robust young European, after witnessing the death of six brother carpenters in the house in which he lived, was attacked about two o'clock in the morning. I saw him a few minutes after the chill had yielded to a fever of the highest grade. In the fever which had been prevailing for some time in Opelousas in the year 1837, active purgatives, mercurials in every form and mode of use, alterative medicines, with all the adjuvants, had hitherto failed. I bled this man to over forty ounces, with some relief to him—gave him a mild purge and left. Very uneasy as to the effect of blood-letting, then very generally condemned in New Orleans, I went back to the patient before sunrise. I found him pale and haggard, but in a profound sleep. Soon I discovered that the bandage had slipped from the orifice in the vein, and that he lay actually soaked in his blood, which had also passed through the mattress and formed a puddle on the floor. After this, not an untoward symptom occurred. He recovered rapidly, taking only some brandy and water, with some mild saline purgative. I have never known debility or congestion to follow an early bleeding, no matter how far pushed. The most timid and inexperienced need fear no such result.

I was called in 1854 to see Mr. A., aged about twenty-eight, extremely robust. He lived in the center of the locality in which the fever raged. Some three hours had elapsed since he was attacked, and he had taken a dose of castor oil. The perspiration streamed from every pore, and though the fever was high, he expressed himself somewhat relieved. With good nursing, and an occasional soda powder, he made a quick recovery.

After the adoption of blood-letting in 1837, a marked diminution of deaths ensued. In 1839, an all-pervasive epidemic visited Opelousas. The fever in all instances was intense, and accompanied with severe pains. Blood-letting freely employed at the onset of the fever seemed an indispensable and certain means of restoration to health. The motality* was as small as was ever seen in any

*In the April number of the Record, page 196, eleventh line from top, *minimum* should have been printed instead of *maximum*.

epidemic embracing an equal number of cases. The employment of blood-letting on the second, and much more so on the third day, is unjustifiable; fatal results are thus likely hastened; and in cases tending toward recovery, the effect may be, as has already been stated, to enfeeble or arrest the salutary actions of the health-power.

This article has been extended far beyond my original intention. Its perusal will satisfy the reader that my incentive in writing has been to elicit the attention of the profession to a further study of the physical phenomena of febrile diseases, and especially of the yellow fever. Whether or not I shall have succeeded, the motive which has actuated me will be felt a sufficient reward for the labor bestowed.

